# Psychosocial distress in people with overweight and obesity: the role of weight stigma and social support

**DOI:** 10.3389/fpsyg.2024.1474844

**Published:** 2025-01-07

**Authors:** Vladimira Timkova, Pavol Mikula, Iveta Nagyova

**Affiliations:** Department of Social and Behavioural Medicine, Faculty of Medicine, PJ Safarik University in Kosice, Kosice, Slovakia

**Keywords:** overweight, obesity, weight stigma, social support, psychosocial distress

## Abstract

We aimed to assess the role of weight stigma and social support in depression, anxiety, and loneliness controlling for sociodemographic and clinical variables. A total of 189 adults with overweight/obesity were included. Participants were recruited from outpatient clinics by general practitioners which covered all regions of Slovakia. Correlation analyses and multiple linear regression were used to analyze the data. Participants experienced weight-related teasing (40.4%), unfair treatment (18.0%), and discrimination (14.1%). We found an association between lower age, female sex and psychological distress. No role of obesity indicators in psychosocial distress was identified, except for a small association between body mass index and depression in correlation analyses. Significant associations between experienced weight stigma/self-stigmatization and psychosocial distress weakened when variables related to the social support system were added to the linear regression. Poor social support was strongly associated with depression, anxiety, and loneliness. The explained variance in the final regression models was 42, 44, and 54%, respectively. Weight stigma negatively affects mental health and a sense of belonging while it seems to be a more significant contributor to psychosocial distress compared to obesity per se. Interventions targeting weight-related self-stigmatization and social relationships may mitigate the negative impact of weight stigma on psychosocial well-being.

## Introduction

Overweight/obesity is defined as abnormal or excessive fat accumulation that represents a health risk. In 2019, an estimated 5 million noncommunicable disease deaths were caused by higher-than-optimal body mass index (BMI). Rates of overweight and obesity continue to grow. From 1990 to 2022, the percentage of adults living with obesity more than doubled from 7 to 16% (WHO, 2024). This increase cannot be solely explained by sudden changes in our genetic background and has been mainly attributed to an obesogenic environment. Obesity is a complex and multifaceted public health problem caused by genetic, and biopsychosocial factors and environments that place individuals in frequent proximity to an abundance of highly caloric foods, thereby eroding self-control resources (Rosenbaum and White, 2016; Albuquerque et al., 2017).

Efficient weight and health management requires a multidisciplinary approach, both at the level of prevention and treatment. Interventions should aim at eating behavior and behavioral programs to increase physical activity and reduction of sedentary activities (Bischoff et al., 2017). Psychological distress and loneliness represent another relevant factor associated with poor quality of life in people with larger body size (Jung and Luck-Sikorski, 2019; Mannucci et al., 2010). Psychosocial interventions seem to be particularly relevant in this context, as they may help to address the emotional and behavioral patterns contributing to a better quality of life and more efficient weight management (Vallis, 2016; van Zyl et al., 2020).

However, efforts to mitigate the epidemic of obesity are often based on weight-centric paradigms and approaches and thus may contribute to the stigmatization of people with overweight and obesity (Hagan and Nelson, 2023; Major et al., 2014; McEntee et al., 2023). As weight is typically assumed to be controllable and easily maintained through diet and physical activity (Hunger et al., 2020), the inability to manage body size/shape is often perceived as a personal moral failure (Ringel and Ditto, 2019). These weight-centric paradigms may lead to harmful assumptions about the lifestyle choices and personalities of people with obesity (Westbury et al., 2023). They are often perceived by the general public or even in clinical settings as lazy, weak, unmotivated, with low self-control, noncompliant, and unhealthy (Cohen and Shikora, 2020; Olson et al., 2023; Phelan et al., 2014; Steptoe and Frank, 2023).

Larger body size is associated with physical comorbidities, but it also affects psychosocial distress, social isolation, and feelings of rejection (e.g., Rotenberg et al., 2017) that may be caused by stigmatization (Hajek et al., 2021). Several studies including systematic reviews and meta-analyses showed that psychological distress and loneliness were found to be associated with morbidity and all-cause mortality, and many chronic diseases such as increased cardiovascular risk (Rico-Uribe et al., 2018; Krittanawong et al., 2023), decreased physical activity (Pels and Kleinert, 2016), poor sleep quality (Craven and Fekete, 2022), and fatigue (Powell et al., 2022). Furthermore, these factors play a role in the development of obesity and may hinder weight control efforts. Obesity was also found to be associated with lower emotional trust in significant others, lower disclosure, and higher loneliness (Rotenberg et al., 2017). However, the role of weight stigma was not assessed in this study.

Weight stigma is defined as negative weight-related attitudes or beliefs, expressed as stereotypes, prejudice, and even open discrimination toward individuals because of their weight (Cohen and Shikora, 2020). It presents across various settings and emerges on individual, interpersonal, institutional, and societal levels (McEntee et al., 2023). Weight stigma is prevalent, pervasive, and often perceived as socially acceptable (Phelan et al., 2014). In Western countries, rates of experienced weight stigma in adults with overweight and obesity vary between 42 and 60% (Lee et al., 2021; Puhl et al., 2021). Inducing shame to promote weight loss is frequently accepted as a valid and well-meaning behavior (Hunger et al., 2020). However, weight stigma was found to have no motivating effects (Wu and Berry, 2018) and may in turn diminish weight control efforts (Steptoe and Frank, 2023). Studies show that weight stigma may even trigger an obesogenic process (Tomiyama et al., 2018), and was found to be associated with poor self-regulation or eating disturbances including overeating (Major et al., 2014; Puhl et al., 2021).

Stigmatization of one’s weight may lead to numerous adverse health consequences depression and anxiety (Steptoe and Frank, 2023; Hayward et al., 2018; Puhl et al., 2021; Wu and Berry, 2018). Chronic stress underlying weight stigma is reported to be associated with a 60% increased risk of mortality not explained by body mass index (BMI) or other clinical and behavioral risk factors (Sutin et al., 2015).

Experiences of stigma and discrimination can become internalized, with individuals of high body weight seeing themselves as to blame for being overweight or obese (Kahan and Puhl, 2017). Moreover, the adverse health consequences were found to be especially strong in people with internalized weight-stigmatizing beliefs (Hayward et al., 2018; Pearl and Puhl, 2018). Self-stigmatization was found to be associated with higher depression, anxiety, and loneliness (Hayward et al., 2018; Forbes and Donovan, 2019; Emmer et al., 2020; Puhl et al., 2021).

So far, studies systematically contextualizing evidence regarding the association between obesity, stigma, social isolation, and loneliness are lacking and the results of systematic reviews are not clear (e.g., Hajek et al., 2021). Little is known about the associations and underlying mechanisms between weight stigma and psychological health outcomes in people with overweight and obesity (Emmer et al., 2020; Wu and Berry, 2018) as previous research focused mainly on the relationship between weight and health. Thus, to identify efficient interventions there is a need for a better understanding of how experienced and internalized weight stigma may affect physiological, psychological, and social outcomes (e.g., Roberts et al., 2021).

The current review and meta-analysis showed a lack of research on protective factors against weight stigma including the role of social support (Emmer et al., 2020). The term “social support” has been often used to describe the mechanisms by which interpersonal relationships presumably buffer one against a stressful environment (Cohen and Wills, 1985). For example, lower emotional support was also found to be associated with the internalization of weight stigma (Himmelstein et al., 2020). However, systematic reviews and meta-analyses on the role of social support in the associations between stigmatization and loneliness, social isolation in overweight/obesity reported inconclusive and incomplete evidence (Hajek et al., 2021). Thus, further research is needed to identify the potential effects of social support on the association between weight stigma and psychosocial distress (Emmer et al., 2020; Hajek et al., 2021). Thus, we aimed to assess the role of weight stigma and social support in depression, anxiety, and loneliness controlling for sociodemographic and clinical variables among people with overweight and obesity.

## Materials and methods

### Participants

We included 189 participants with overweight and obesity (52.9% female; mean age 48.8 ± 14.5 years; body-mass index 32.6 ± 6.5 kg/m^2^; waist-to-height ratio 0.6 ± 0.1). Participants were recruited from outpatient clinics by general practitioners (GPs) which covered all regions of Slovakia. There was no exclusion of participants based on comorbidities. The participants were included based on overall overweight and obesity (using BMI) and central obesity (using WHtR) scores. Exclusion criteria were a BMI of ≤24.99, and a WHtR of ≤0.49 which is not associated with central obesity or health risks (Ashwell and Gibson, 2016), the inability to speak the Slovak language, and being <18 years of age.

### Procedure

Data collection in this cross-sectional study consisted of questionnaires and measurements of participants’ height, weight, and waist circumference. Questionnaires were translated from the English language to the Slovak language following the standard procedure (Grégoire and Itc, 2018). First, two independent bilingual translators, one also an expert in psychology translated the questionnaires. Then, a forward-backwards translation was conducted. To optimize the translation, differences between the original and the back translation were discussed by the research team. Participants filled in the questionnaires at their own pace at home. Informed consent was signed by all participants included in the study. Participation in the study was voluntary. Incentives in the form of financial vouchers were offered for participation in the research. The Ethics Committee of Pavol Jozef Safarik University (EK 1 N/2023) approved the study. All procedures performed in studies involving human participants were following the ethical standards of the institutional and/or national research committee and with the 1964 Helsinki Declaration and its later amendments or comparable ethical standards. Data were collected between May 2023 and December 2023.

### Measures

#### Psychosocial distress

*The 9-item Self-report Patient Health Questionnaire (PHQ-9)* was used to identify the presence of depressive symptoms based on the nine DSM-IV criteria for major depressive disorder. Respondents indicated how frequently (0 = “not at all”; 3 = “nearly every day”) they have been bothered by the problems listed. Each item is rated using a four-point Likert scale, with scores ranging from 0 to 27. The higher scores represent greater depression, with the cut-off scores for mild, moderate, moderately severe, and severe depressive symptoms of 5, 10, 15, and 20, respectively (Kroenke et al., 2001). The Cronbach’s alpha in our sample was 0.88.

*We used the* 7-item *General Anxiety Disorder Questionnaire (GAD-7)* to assess anxiety. Items are rated on a 4-point Likert-type scale (0 = “not at all”; 3 = “nearly every day”) and describe some of the most salient diagnostic features of GAD (i.e., *feeling nervous, anxious, or on edge and worrying too much about different things*). A total score range from 0 to 21, with higher scores indicating higher anxiety. For mild, moderate, and severe anxiety symptoms, the cut-off values are 5, 10, and 15, respectively (Spitzer et al., 2006). Cronbach’s alpha in our sample was 0.94.

A revised 20-item *UCLA Loneliness Scale was* designed to measure one’s subjective feelings of loneliness as well as feelings of social isolation (Russell et al., 1980). Participants rated each item on a scale from 1 (never) to 4 (often). A total of 10 of the 20 original items are scored. The range of potential scores is from 20 to 80 with higher scores indicating a higher level of loneliness. The cut-offs for loneliness severity were adapted from Miaskowski et al. (2021) as follows: none or minimal (scores of 20–35), moderate (scores of 36–49), moderately high (scores of 50–64), and high level of loneliness (scores of 65 and above). Cronbach’s alpha in our sample was 0.85.

### Experienced weight stigma

*The Experienced Weight Stigma (EWS) Questionnaire* (Puhl et al., 2017) assesses the previous history of experienced stigma. It consists of three yes (=1) /no (=2) questions that ask participants if they have ever “*(1) been teased/ (2) treated unfairly/ (3) discriminated against because of their weight?”* Lower scores indicate a higher level of experienced stigma with answers summed to create a total experienced stigma scale that ranged from 3 (experienced all three types of stigmatization) to 6 (never experienced weight stigmatization). To identify individuals who experienced weight stigma we dichotomized variables based on answered “yes” to at least one of the three items. Cronbach’s alpha in our sample was 0.79.

#### Weight self-stigma

*The Weight Self-Stigma Questionnaire* (WSSQ) is a 12-item measure of weight-related self-stigma (Lillis et al., 2010). WSSQ items were rated on a scale of 1 (completely agree) to 5 (completely disagree). Items 1–6 constituted the *self-devaluation subscale*, and items 7–12 constituted the *fear of enacted stigma* subscale. Sum scores can be calculated for the full scale and each subscale. The scale may range from 12 to 60 with a higher score indicating lower weight self-stigmatization. Cronbach’s alpha in our sample for the self-devaluation subscale was 0.89 and 0.87 for the fear of enacted stigma subscale. Cronbach’s alpha in our sample was 0.91 for the overall scale.

#### Social support

S*ocial support* was assessed using the Oslo Social Support Scale (OSSS-3). It consists of three items that ask about the *number of close confidants* (ranging from 1 = “none” to 4 = “more than five”), *the sense of concern from other people* (ranging from 1 = “none” to 5 = “a lot”), and the *relationship with neighbors with a focus on the accessibility of practical help* (ranging from 1 = “very difficult” to 5 = “very easy”; Kocalevent et al., 2018). The sum score ranges from 3 to 14, with higher values representing higher social support. The score between 3 and 8 indicated poor social support; scores between 9 and 11 indicated a moderate level of social support; and scores between 12 and 14 indicated strong social support. Cronbach’s alpha in our sample was 0.67. The internal consistency of the OSSS-3 with *α* = 0.64 could be regarded as acceptable (Kocalevent et al., 2018).

Another scale used to address variables related to the social support system was *the Family APGAR Scale* designed to test five areas of family function such as adaptation, partnership, growth, affection, and resolve (Smilkstein et al., 1982). This scale is used to diagnose dysfunction in the family system and help perform interventions to balance family relationships (Karimi et al., 2022). Responses were recorded on a 3-point scale (from 0=” hardly ever” to 2=” almost always”), with higher scores indicating better family functioning. The total scores are categorized as follows: 7–10 indicating good family functioning, 4–6 indicating moderate family functioning, and 0–3 severely dysfunctional family functioning (Smilkstein et al., 1982). Cronbach’s alpha in our sample was 0.89.

#### Sociodemographic and clinical variables

Sociodemographic data were collected using a questionnaire, with some variables assessed as categorical variables (sex, education, place of residence, and relationship status) and others as continuous (age and household income). Data on weight, height, and waist circumference were assessed by healthcare professionals. The measures were taken barefoot with indoor clothes using an electronic scale for body weight, tape, and a wall-mount stadiometer. We calculated BMI in kg/m^2^. The waist-to-height ratio (WHtR) was used to assess central obesity. The WHtR of each participant was obtained by dividing waist circumference by height.

### Statistical analyses

Statistical analyses consisted of descriptive analyses of all key variables under study, followed by correlation analyses, and multiple linear regression analysis (enter method) to assess the explained variance of psychosocial distress domains measured as depression, anxiety, and loneliness. The order of variables included in the regression models was as follows: 1. age, sex, 2. education, household income, place of residence, relationship status 3. waist-to-height ratio (WHtR), 4. experienced weight stigma, 5. weight self-stigma, and 6. family function, and social support. Power analysis revealed (*n* = 189) that the statistical power for multivariate analysis exceeds 88% at *α* = 0.05 and medium effect size (Faul et al., 2009). All analyses were performed using the Statistical Package for the Social Sciences (IBM SPSS 26). Multicollinearity was assessed using the variance inflation factor (VIF < 2.0). A *p*-value of <0.05 was considered statistically significant.

## Results

The basic description of the sample is shown in Table 1 (*n* = 189). The participants averaged 48.8 ± 14.5 years; 52.9% were female with a mean BMI of 32.6 ± 6.5 kg/m^2^ and WHtR of 0.6 ± 0.1. A total of 6.9% of participants had both overall obesity and central obesity with a severe risk of weight-related health problems. Participants experienced weight-related teasing (40.4%), unfair treatment (18.0%), and discrimination (14.1%). At least one of these events was reported by 41.0% of participants. We found that 52.0% of participants in our study experienced mild to severe depressive symptomatology, 39.9% reported mild to severe symptoms of anxiety, and 50.0% experienced loneliness.

**Table 1:** Characteristics of the study population (*n* = 189).

Variables	Mean ± SD/%
Age in years; mean ± SD	48.8 ± 14.5
Sex; female (%)	52.9%
Education	
Elementary	5 (2.7%)
Secondary	97 (52.2%)
University	84 (45.2%)
Ethnicity; white (%)	184 (97.4%)
Relationship status; single (%)	54 (28.9%)
Household income (Eur)*	1960 ± 1,170
Place of residence; rural/town**	110 (60.1%)
Body mass index (BMI); mean ± SD	
Overweight (25.00–29.99)	73 (38.6%)
Obese (≥30.00)	116 (61.4%)
Waist-to-height-ratio (WHtR)	
WHtR with increased risk of health problems (0.50–0.59)	176 (93.1%)
WHtR with severe risk of health problems (≥0.60)	13 (6.9%)
Experienced Weight Stigma (EWS; 3–6); mean ± SD	5.3 ± 1.0
Experienced weight stigma (yes)	41.0%
Experienced teasing	40.4%
Experienced unfair treatment	18.0%
Experienced discrimination	14.1%
Weight Self-stigma; (WSS; 12–60); mean ± SD	42.3 ± 11.2
Fear of enacted stigma	23.13 ± 5.93
Self-devaluation subscale	19.22 ± 6.56
Oslo Social Support Scale; (OSS-3; 3–14); mean ± SD	10.5 ± 2.2
Poor social support (3–8)	26 (19.1%)
Moderate social support (9–11)	64 (47.1%)
Strong social support (12–14)	46 (33.8%)
Family APGAR Scale; (APGAR; 0–10); mean ± SD	8.4 ± 2.4
Good family functioning	75.8%
Moderate family functioning	20.3%
Low family functioning	3.9%
Patient health Questionaire (PHQ-9; 0–27); mean ± SD	6.1 ± 4.8
None to minimal depression (0–4)	72 (48.0%)
Mild depression (5–9)	47 (31.3%)
Moderate depression (10–14)	21 (14.0%)
Moderately severe depression (15–19)	6 (4.0%)
Severe depression (20–27)	4 (2.7%)
General Anxiety Disorder Questionnaire (GAD-7; 0–21); mean ± SD	4.4 ± 4.1
None to minimal anxiety (0–4)	92 (60.1%)
Mild anxiety (5–9)	43 (28.1%)
Moderate anxiety (10–14)	14 (9.2%)
Severe anxiety (15–21)	4 (2.6%)
UCLA Loneliness Scale (UCLA; 20–80); mean ± SD	36.1 ± 8.4
None to minimal loneliness (<36)	72 (50.0%)
Moderate loneliness (36–49)	61 (42.4%)
Moderate high loneliness (50–64)	11 (7.6%)
High loneliness (≥65)	0 (0%)

*Household income netto per month; **Place of residence: Kosice and Bratislava were considered as cities (Buzalka, 2023). Missing values: Education: (1.6%); Relationship status: (1.1%); Household Income (0.5%); Place of living (3.2%); OSS (29.9%); GAD (19.0%); PHQ (20.6%); UCLA (23.8%); EWS (3.1%); WSS (23.7%); APGAR (21.1%); For the lowest number of participants included in the OSSS-3 subscale (n = 136), the statistical power for multivariate analysis exceeds 85% at α = 0.05 and medium effect size (Faul et al., 2009); *p < 0.05; **p ≤ 0.01; ***p ≤ 0.001.

### Correlation analyses

We found a significant association between lower age (*r* = 0.30; *p* ≤ 0.001), being female (−0.15; *p* < 0.05) and experiencing weight stigma. No significant role of sociodemographic variables was observed in association with self-stigmatization, however. Obesity indicators were significantly associated with experienced weight stigma and self-stigmatization. Self-stigmatization was strongly associated with experienced weight stigma (*r* = 0.51; *p* ≤ 0.001). Experienced stigma and self-stigmatization were both significantly associated with higher family dysfunction (*r* = 0.26; *p* ≤ 0.01; *r* = 0.30; *p* ≤ 0.001) and poor level of social support (*r* = 0.26; *p* ≤ 0.01; *r* = 0.39; *p* ≤ 0.001), respectively. We also found higher WHtR (*r* = −0.26; *p* ≤ 0.01) and BMI (*r* = −0.29; *p* ≤ 0.001) to be significantly associated with family dysfunction.

No significant role of sociodemographic variables and obesity indicators was observed in association with psychosocial distress, except for a small but significant role of younger age (*r* = −0.18; *p* < 0.05) and higher BMI (*r* = 0.18; *p* < 0.05) in depression and female sex (*r* = 0.21; *p* < 0.05) in higher anxiety. Experienced weight stigma and weight self-stigmatization were strongly associated with all assessed indicators of psychosocial distress (*p* ≤ 0.001), except for a small association between experienced weight stigma and loneliness (−0.21; *p* < 0.05). Higher social support and family function were strongly associated with lower psychosocial distress (*p* ≤ 0.001). More details are depicted in Table 2.

**Table 2:** Correlation analyses of the associations between sociodemographic and clinical variables, psychological distress, and weight stigma.

	Age	Sex	Educ	Income	Resid	WTHR	BMI	RS	EWS	WSS	Self_dev	Fear_stig	APGAR	OSS	PHQ	GAD
Sex	0.06															
Education	−0.25***	0.13														
Income	−0.18*	−0.22**	0.24**													
Resid	0.05	0.04	0.23**	−0.02												
WHtR	0.24**	0.07	−0.14	−0.13	−0.07											
BMI	0.03	0.10	−0.12	−0.09	0.00	0.68***										
RS	0.03	−0.15*	−0.02	0.39***	−0.18*	0.03	−0.07									
EWS	0.30***	−0.15*	−0.13	0.05	−0.09	−0.16*	−0.38***	0.07								
WSS	0.06	−0.11	0.07	0.02	−0.01	−0.26**	−0.46***	−0.02	0.51***							
Self_dev	0.01	−0.12	0.03	−0.03	0.00	−0.17*	−0.38***	−0.03	0.39***	0.92***						
Fear_stig	0.10	−0.04	0.11	0.07	−0.04	−0.29***	−0.44***	0.02	0.53***	0.89***	0.61***					
APGAR	0.01	−0.17*	−0.01	0.17*	−0.02	−0.26**	−0.29***	0.00	0.26**	0.30***	0.22**	0.31***				
OSSS	0.15	−0.03	−0.01	0.01	0.02	−0.08	−0.08	−0.10	0.26**	0.39***	0.31***	0.37***	0.35***			
PHQ	−0.18*	0.14	−0.04	−0.13	−0.04	0.08	0.18*	−0.04	−0.43***	−0.50***	−0.42***	−0.49***	−0.36***	−0.50***		
GAD	−0.12	0.21*	−0.10	−0.09	−0.12	0.02	0.13	0.01	−0.32***	−0.40***	−0.36***	−0.36***	−0.33***	−0.46***	0.75***	
UCLA	−0.00	0.11	−0.05	−0.12	−0.08	0.06	0.09	0.04	−0.21*	−0.41***	−0.31***	−0.42***	−0.52***	−0.69***	0.55***	0.54***

Resid, Place of residence; WHtR, waist to height ratio; BMI, Body Mass Index; RS, Relationship Status; EWS, experienced weight stigma; WSS, weight self-stigma; Self_dev, Self devaluation subscale of WSSQ; Fear_stig, Fear of enacted stigma subscale of WSSQ; APGAR, APGAR family function; OSSS, Oslo social support Scale; *p < 0.05; **p ≤ 0.01; ***p ≤ 0.001. Bold values indicate significance.

### Regression analyses

#### The associations of sociodemographic and clinical variables with psychosocial distress

Regression analyses showed an association between lower age (*β* = −0.30; *p* < 0.01) and depression. Lower age (*β* = −0.27; *p* < 0.01) and female sex (*β* = 0.25; *p* < 0.01) were significantly associated with anxiety. We found no association between education, income, place of residence, or relationship status with psychosocial distress. No role of waist-to-height ratio (WHtR) in experienced anxiety (*β* = 0.08; *p* > 0.05), depression (*β* = 0.14; *p* > 0.05), or loneliness (*β* = 0.16; *p* > 0.05) was identified. We found no association between body mass index (BMI), anxiety (*β* = 0.16; *p* > 0.05), depression (*β* = 0.16; *p* > 0.05), or loneliness (*β* = 0.15; *p* > 0.05) using additional regression analyses to control for overall obesity.

#### The associations of weight stigmatization with psychosocial distress

Significant associations were found between experienced weight stigma, anxiety (*β* = −0.36; *p* < 0.01), and depression (*β* = −0.44; *p* < 0.001). However, these associations weakened when self-stigmatization was added to the regression models. The association between experienced stigma and loneliness (*β* = −0.27; *p* < 0.05) was only weak and no longer significant (*β* = −0.06; *p* > 0.05) when weight self-stigma was added to the regression model.

Weight self-stigmatization was significantly associated with anxiety (*β* = −0.28; *p* < 0.01), and depression (*β* = −0.37; *p* < 0.01). The strongest association was found between self-stigmatization and loneliness (*β* = −0.44; *p* < 0.001). Additional regression analyses showed that fear of enacted stigma and self-devaluation subscales of self-stigmatization were strongly associated with depression (*β* = −0.28; *p* < 0.01; *β* = −0.29; *p* < 0.01) and loneliness (*β* = −0.39; *p* < 0.01; *β* = −0.35; *p* < 0.001), respectively. The association between the self-devaluation subscale and anxiety (*β* = −0.22; *p* < 0.05) was only weak. No association was found between fear of enacted stigma subscale and anxiety (*β* = −0.20; *p* > 0.05).

#### The associations of social support systems with psychosocial distress

Associations between experienced stigma/self-stigmatization and depression (*p* < 0.01) weakened (*p* < 0.05) when social support was added to the final regression model. No role of family dysfunction in depressive symptomatology was observed using regression analyses.

The association between weight stigma and anxiety was no longer significant when family function (*β* = −0.36; *p* < 0.001) and social support (*β* = −0.25; *p* < 0.01) were added to the regression model. Family function and social support were significantly associated with loneliness and seemed to diminish the negative role of self-stigmatization in the sense of belonging. The total explained variance in the final regression models for depression, anxiety, and loneliness was 42, 44, and 54%, respectively. Detailed results of regression analyses are shown in Table 3.

**Table 3:** Regression analyses of depression, anxiety, and loneliness on sociodemographic and clinical variables, experienced stigma, self-stigma, family function, and social support.

		Depression		Anxiety		Loneliness	
		R^2^	Beta	R^2^	Beta	R^2^	Beta
Model 1	Age	0.09	−0.30**	0.11**	−0.27**	−0.01	−0.04
	Sex		0.15		0.25**		0.07
Model 2	Age	0.07	−0.33	0.12	−0.32**	0.02	−0.11
	Sex		0.13		0.27**		0.06
	Education		−0.22		−0.08		−0.04
	Income		−0.15		−0.06		−0.18
	Place of residence		0.01		−0.08		−0.10
	RS		0.08		0.15		0.22
Model 3	Age	0.08	−0.37**	0.11	−0.34**	0.03	−0.15
	Sex		0.12		0.27**		0.06
	Education		0.01		−0.07		−0.01
	Income		−0.13		−0.05		−0.17
	Place of residence		0.16		−0.08		−0.09
	RS		0.08		0.15		0.23
	WHtR^†^		0.14		0.08		0.16
Model 4	Age	0.23***	−0.17	0.21*	−0.18	0.08*	−0.03
	Sex		0.09		0.23*		0.04
	Education		−0.03		−0.10		−0.05
	Income		−0.13		−0.05		−0.16
	Place of residence		−0.02		−0.10		−0.10
	RS		0.09		0.15		0.22
	WHtR		−0.02		−0.04		0.06
	EWS		−0.44***		−0.36**		−0.27*
Model 5	Age	0.32***	−0.15	0.25**	−0.16	0.20***	−0.02
	Sex		0.07		0.21*		−0.01
	Education		0.01		−0.08		0.02
	Income		−0.16		−0.07		−0.21
	Place of residence		−0.05		−0.12		−0.13
	RS		0.10		0.15		0.24
	WHtR		−0.13		−0.13		−0.05
	EWS		−0.28**		−0.24*		−0.06
	WSS		−0.37**		−0.28**		−0.44***
Model 6	Age	0.42***	−0.08	0.44***	−0.07	0.54***	0.10
	Sex		0.03		0.14		−0.05
	Education		0.02		0.02		0.01
	Income		−0.11		−0.05		−0.10
	Place of residence		−0.04		0.02		−0.11
	RS		−0.01		−0.12		0.07
	WHtR		−0.14		−0.02		−0.00
	EWS		−0.22*		−0.13		−0.01
	WSS		−0.24*		−0.16		−0.20*
	APGAR		−0.17		−0.36***		−0.24**
	OSS		−0.29**		−0.25**		−0.54***

R^2^, Adjusted R Square; RS, Relationship status: single was set as a reference; WHtR, Waist/Height Ratio; Place of residence: rural/town was set as a reference; Sex: a male was set as a reference; EWS, Experienced Weight Stigma; WSS, Weight Self-stigma; APGAR, APGAR Family Function; OSS, Oslo social support Scale; ^†^we used only WHtR due to multicollinearity with BMI; *p < 0.05; **p < 0.01; ***p < 0.001. Bold values indicate significance.

## Discussion

We aimed to assess the role of weight stigma and social support in depression, anxiety, and loneliness controlling for sociodemographic and clinical variables. Participants in our study experienced weight-related teasing (40.4%), unfair treatment (18.0%), and discrimination (14.1%). At least one of these events was reported by 41.0% of participants. This number is consistent with previous research conducted in Western countries with reported rates of experienced weight stigma in adults with overweight and obesity that varied between 42 and 60% (Lee et al., 2021; Puhl et al., 2021).

We also found a significant association between lower age, female sex, and obesity indicators with experienced weight stigma using correlation analyses. Current findings also suggest that being older and male is related to less frequent body-shaming remarks (Spahlholz et al., 2016; Thompson and Bardone-Cone, 2019). Self-stigma was found to be associated with larger body size while no role of sociodemographic variables in weight self-stigmatization was observed in our study. All in all, these findings indicate that women and younger people may experience weight stigma more often when compared to men and people of higher age. However, the experience of weight stigma seems to have no further effect on the internalization process of stigma within these groups. Based on the revealed association between weight stigma, family dysfunction and body size we may also hypothesize that weight stigma and discrimination not only contribute to psychosocial distress but may also mitigate weight control efforts (e.g., Steptoe and Frank, 2023). Longitudinal studies are needed to confirm causality, however.

Findings of multiple linear regression also suggest younger people with overweight and obesity may experience higher levels of anxiety and depression. Anxiety was found to be also higher in females. In line with our findings, no association was identified between depression and female sex in people with obesity in the current studies (Heidari-Beni et al., 2021; Sharafi et al., 2020). Previous research on people with overweight and obesity also showed an association between anxiety and female gender (Heidari-Beni et al., 2021; Sharafi et al., 2020), especially in women with abdominal obesity (Heidari-Beni et al., 2021). Anxiety may be caused by higher social pressure on women to be thin when compared to men (Sharafi et al., 2020), as well as more common experience of stigmatization or weight-related discrimination in females (Spahlholz et al., 2016) as confirmed also in our study. Anxiety may also stem from intrapersonal conflict—food planning and providing often take a large portion of a woman’s day (Koster et al., 2022). At the same time, societal pressure to maintain a thin body is present and often promotes restrictive eating behaviors and dieting (Odgen, 2010; Prnjak et al., 2020).

We found no role of having a partner, place of residence, education, or household income in experiencing psychosocial distress. We may assume that having a partner may have a buffering effect on psychological distress and loneliness, however, various factors may play a role; e.g. mixed-weight couples may experience even more conflict when compared to matched-weight couples (Burke et al., 2012). The negative effect of weight-based discrimination on psychological well-being was found to be associated with social status in the previous study (Ciciurkaite and Perry, 2018). Psychological distress due to weight-related discrimination in women with the lowest household income was found to be significantly higher when compared to women with higher household income. These results suggest that higher household income and social status may have a buffering effect on the association between weight stigma and psychological well-being (Ciciurkaite and Perry, 2018). No significant role of income in our study may be explained by small differences in salaries as Slovakia has one of the lowest levels of income inequality globally (World Bank, 2021), with simultaneously high prices and relatively low salaries (EUROSTAT, 2024). Future research with a larger study sample is needed though to shed more light on the role of income and social status, e.g., access to more expensive quality food with better nutritious values may play a role in lower experienced psychological distress (AlAmmar et al., 2020).

We found no significant association between WHtR/BMI and depression, anxiety, or loneliness using multivariate analyses. Only a small association between BMI and depression was observed using correlation analyses. A recent large meta-analysis of 105 studies (Emmer et al., 2020) also found a significant association between perceived obesity stigma and poorer mental health, which remained significant following adjustment for relevant contributors including body weight measurements. The weight-centric paradigms often present higher weight as a precursor to poor physical health and psychosocial distress (e.g., Hunger et al., 2020). However, our findings suggest that psychological distress may be caused by weight stigmatization rather than by overweight/obesity per se. Thus, our findings of the limited role of body size in psychosocial distress in people with overweight and obesity may help to foster more empathy in weight and health management.

Our results suggest a significant role of experienced stigma in psychosocial distress. However, the association between experienced stigma and psychosocial distress weakened or became insignificant when weight self-stigmatization was added to the regression models. All in all, it seems that weight self-stigmatization may play a more significant role in depression, anxiety, and loneliness when compared to experienced stigma. In line with our findings, a systematic review of 74 studies found links between weight bias internalization and greater depression, anxiety, and lower health- and mental health-related quality of life (Puhl et al., 2021). A current meta-analysis (Emmer et al., 2020) also described a more significant role of self-stigmatization in poor mental health outcomes when compared to the role of experienced stigma. These larger effects of internalized stigma may be a consequence of the acceptance of prejudice and negative stereotypes being true for oneself (Emmer et al., 2020). Hence, weight stigma becomes relevant for the self, which may impact mental health in a more significant way. Furthermore, it may be more difficult to escape stigma-related distress once weight stigmatization is internalized (Emmer et al., 2020). Thus, it seems that interventions targeting weight-related self-stigmatization may diminish the also negative impact of experienced weight stigma on psychosocial well-being.

A total of 50.0% of participants in our study experienced an increased level of loneliness. This number is very high as according to a current meta-analysis the prevalence of loneliness in the general population varies between 1.8 to 6.5% in northern European countries and 5.9 to 24.2% in eastern European countries (Surkalim et al., 2022). Our study also showed a strong association between self-stigmatization and loneliness. Surprisingly, the association between experienced stigma and loneliness was only weak and no longer significant when self-stigmatization was added to the regression model. Our findings further indicate that people with higher internalized weight bias, especially those with fear of enacted stigma may experience loneliness more keenly.

We found that 39.9% of participants reported increased anxiety and 52.0% of participants in our study experienced mild to severe depressive symptomatology. A total of 20.7% of participants in our study reported moderate to severe depression based on the criteria for major depressive disorder. The American Psychiatric Association reported that less than 10% of people with obesity are likely to meet the full criteria for a major depressive disorder (Bray and Wadden, 2020). Although it seems that depression may be higher in our sample, we should consider that it may be due to cross-cultural differences. For example, people in Slovakia currently report the lowest level of life satisfaction in Europe (Slovak Academy of Sciences, 2024). Another explanation may be the negative influence of the ongoing war in the neighboring country that was found to increase psychological distress (Chudzicka-Czupała et al., 2023).

The encouraging findings of our research are that it seems that interventions targeting social support systems may mitigate the negative impacts of weight stigma on psychological well-being and sense of belonging. The associations between weight stigma and anxiety were no longer significant when social support and family function were added to the final regression model. Not surprisingly, higher social support was associated especially strongly with lower levels of loneliness. Social support was able to diminish the association between stigma and depression, while no significant role of family function in experienced depressive symptomatology was identified using regression analyses. Correlation analyses showed an association between family dysfunction and depression while also indicating the association between weight stigma and perceived family dysfunction. Thus, it seems that stigma may play a significant role in the association between family function and depression. However, future studies are needed to shed more light on the role of family environment as a possible source of stigma. Previous studies on the role of social support in people with overweight and obesity were inconclusive and some found no buffering effect of social support against the adverse effects of perceived weight stigmatization on mental health (Emmer et al., 2020; Hajek et al., 2021). Thus, future studies are needed to assess the role of different types of social support on the association between weight stigma and psychological distress (Emmer et al., 2020) as well as different sources of stigmatization while considering sociodemographic factors or changes across the lifespan.

### Strengths and limitations

To our best knowledge, only a few studies have assessed the role of social support and family function in the association between weight stigma and psychosocial distress, and those that have yielded inconclusive results. Our findings have the potential to challenge weight-centric paradigms by bringing evidence that other factors may influence the psychological health and sense of belonging of people with larger body sizes independent of body weight. A few limitations have to be considered, however. Because we used cross-sectional data, we were not able to establish causal relationships. Another limitation is a higher number of missing data for some variables due to two different sets of questionnaires used during the data collection. However, also for the lowest number of participants included in the OSSS-3 subscale (*n* = 136), the statistical power for multivariate analysis was still satisfactory and exceeded 85% at *α* = 0.05 and medium effect size (Faul et al., 2009). Our study focused on individuals with overweight/obesity, acknowledging they are more likely to encounter stigma as a by-product of “fat phobia,” societal ideals, and attributions of weight to individual responsibility (Eisenberg et al., 2015). However, it is important to consider that weight stigma and its effects occur across the weight spectrum, and also people with healthy body weight may experience weight stigmatization. For example, a previous study (Eisenberg et al., 2015) found the portrayal of weight stigmatization on popular television shows—including targeting women of average weight. Thus, future studies should address also the experience of individuals with obesity indicators below the overweight threshold. Moreover, the sample of participants of different ethnicities was very small, with four Roma people and one Asian participant. Although our predominantly white sample more or less reflects the general population in Slovakia, we were not able to explore how these associations affect populations of diverse backgrounds. Another limitation is the inability of our study to identify the specific sources of experienced weight stigma and their relative contributions to psychosocial distress. Although we were able to assess the prevalence of experienced weight stigma, the used methodology did not allow for the identification of its various sources, such as stigma from family members, friends, media, or healthcare settings, nor determination of the amount of their impact on the psychological well-being and sense of belonging. For example, we observed that experienced stigma and self-stigmatization were both significantly associated with higher family dysfunction and lower social support. We also found that poor family functioning and low social support played a role in psychological distress and experienced loneliness. Thus, we may assume that weight stigma experienced in the family environment may represent one of the main sources of psychosocial distress (e.g., Lawrence et al., 2022). Due to the cross-sectional nature of our data, we are unable to determine the sources that shape stigma, or how experienced weight stigma and self-stigmatization may affect health-related outcomes and social functioning over time. Thus, it may be important to differentiate between childhood and adult experiences of weight stigma and to identify their potential long-term negative ramifications including their cumulative effect on health.

### Implications for practice and future research

Even though weight stigma is a unique contributor to adverse physical and mental health outcomes (Puhl et al., 2021; Wu and Berry, 2018) it is rarely targeted in prevention and intervention efforts to provide tools to identify, validate, and cope with weight stigma and its internalization (McEntee et al., 2023). More studies with larger study samples are needed to shed light on the role of sociodemographic variables such as age, sex, income, or relationship status including the role of body size discrepancies between partners.

Based on our findings it seems that experienced and perceived weight stigma may negatively affect mental health and sense of belonging following adjustment for relevant contributors including body weight measurements. The adverse consequences were found to be especially strong in people with internalized weight self-stigmatizing beliefs. The encouraging conclusion of our study is that social support systems may help to diminish psychosocial distress caused by weight stigma. Thus, we may assume that interventions targeting weight-related self-stigmatization and social relationships may mitigate also the negative impact of weight stigma on psychosocial well-being. Loneliness could increase the burden and lower overall quality of life in people with larger body sizes. Social withdrawal may also lead to avoidance of medical care including help-seeking behaviors in terms of weight management. It is therefore important to support those who are vulnerable to weight bias by helping them to build up social networks, as social relationships are important not only for physical health but also for emotional and psychological well-being (Jung and Luck-Sikorski, 2019). Thus, cost-effective programs for multidisciplinary care that will include also community and family interaction may be helpful. Based on our findings of the significant association between family dysfunction and experienced weight stigma/self-stigmatization we may assume that the family environment may represent a source of weight bias. We further found that family dysfunction was significantly higher in people with larger body sizes. As dense social networks were also found to be associated with improvement in weight status (Yoon and Brown, 2011), future studies should shed more light on these associations.

All in all, our findings indicate that weight stigma may be a more significant contributor to depression, anxiety, and loneliness than obesity per se. Thus, a more comprehensive approach that integrates biological determinants of adiposity, mental health, social isolation, loneliness, and social support may prove valuable (Emmer et al., 2020; Hajek et al., 2021; Steptoe and Frank, 2023). Clinicians and public health professionals should be the ones who lead the efforts to nudge strategies that may help to eliminate weight stigmatization (Cohen and Shikora, 2020), as they can create a weight-inclusive, non-judgemental, welcoming atmosphere and focus on well-being and health rather than just raw weight loss (Tylka et al., 2014). However, the media, the general public, academic literature, and healthcare professionals still overwhelmingly focus on the contribution of individual choices and responsibility and often blame people for weight-related problems without acknowledging the broader societal, environmental, and systemic factors encouraging obesity (Cohen and Shikora, 2020; Zafir and Jovanovski, 2022). Therefore, a multidisciplinary, focused, and sustained effort from stakeholders and key decision-makers within society is required to dispel myths around personal responsibility for body weight and to foster more empathy for people with overweight and obesity (Westbury et al., 2023). Education about weight stigma as well as policies to protect people against stigmatization is an important challenge for diminished psychosocial distress in people with overweight/obesity. To create more efficient intervention strategies future studies should also identify possible sources of weight stigma and its adverse health and social outcomes over time.

## Conclusion

Experienced weight stigma and self-stigmatization may negatively affect mental health and sense of belonging following adjustment for relevant contributors including body weight measurements. The adverse consequences were found to be especially strong in people with internalized weight self-stigmatizing beliefs. It also seems that adequate social support may diminish psychosocial distress caused by weight stigma. Interventions targeting weight-related self-stigmatization and social relationships may mitigate the negative impact of weight stigma on psychosocial well-being.

## Data Availability

The raw data supporting the conclusions of this article will be made available by the authors, without undue reservation.
